# A new fluorescent sensor mitoferrofluor indicates the presence of chelatable iron in polarized and depolarized mitochondria

**DOI:** 10.1016/j.jbc.2022.102336

**Published:** 2022-08-02

**Authors:** Andaleb Kholmukhamedov, Li Li, Christopher C. Lindsey, Jiangting Hu, Anna-Liisa Nieminen, Kenji Takemoto, Gyda C. Beeson, Chad M. Beneker, Campbell McInnes, Craig C. Beeson, John J. Lemasters

**Affiliations:** 1 Center for Cell Death, Injury & Regeneration, Medical University of South Carolina, Charleston, South Carolina, USA; 2 Department of Drug Discovery & Biomedical Sciences, Medical University of South Carolina, Charleston, South Carolina, USA; 3 Hollings Cancer Center, Medical University of South Carolina, Charleston, South Carolina, USA; 4 Department of Drug Discovery & Biomedical Sciences, College of Pharmacy, University of South Carolina, Columbia, South Carolina, USA; 5 Department of Biochemistry & Molecular Biology, Medical University of South Carolina, Charleston, South Carolina, USA

**Keywords:** iron sensor, mitochondria, membrane potential, ischemia/reperfusion, ΔΨm, mitochondrial membrane potential, CCCP, carbonyl cyanide m-chlorophenyl hydrazone, ER, endoplasmic reticulum, FAS, ferrous ammonium sulfate, FCCP, carbonyl cyanide trifluoromethoxyphenylhydrazone, FCS, fetal calf serum, FeHQ, Fe3+/8-hydroxyquinoline, MFF, mitoferrofluor, MTG, MitoTracker green, OCR, oxygen consumption rate, PIH, pyridoxal isonicotinoyl hydrazone, PMP, plasma membrane permeabilizer, ROS, reactive oxygen species, RPA, rhodamine B-[(1,10-phenanthrolin-5-yl)aminocarbonyl]benzyl ester

## Abstract

Mitochondrial chelatable iron contributes to the severity of several injury processes, including ischemia/reperfusion, oxidative stress, and drug toxicity. However, methods to measure this species in living cells are lacking. To measure mitochondrial chelatable iron in living cells, here we synthesized a new fluorescent indicator, mitoferrofluor (MFF). We designed cationic MFF to accumulate electrophoretically in polarized mitochondria, where a reactive group then forms covalent adducts with mitochondrial proteins to retain MFF even after subsequent depolarization. We also show in cell-free medium that Fe^2+^ (and Cu^2+^), but not Fe^3+^, Ca^2+^, or other biologically relevant divalent cations, strongly quenched MFF fluorescence. Using confocal microscopy, we demonstrate in hepatocytes that red MFF fluorescence colocalized with the green fluorescence of the mitochondrial membrane potential (ΔΨ_m_) indicator, rhodamine 123 (Rh123), indicating selective accumulation into the mitochondria. Unlike Rh123, mitochondria retained MFF after ΔΨ_m_ collapse. Furthermore, intracellular delivery of iron with membrane-permeant Fe^3+^/8-hydroxyquinoline (FeHQ) quenched MFF fluorescence by ∼80% in hepatocytes and other cell lines, which was substantially restored by the membrane-permeant transition metal chelator pyridoxal isonicotinoyl hydrazone. We also show FeHQ quenched the fluorescence of cytosolically coloaded calcein, another Fe^2+^ indicator, confirming that Fe^3+^ in FeHQ undergoes intracellular reduction to Fe^2+^. Finally, MFF fluorescence did not change after addition of the calcium mobilizer thapsigargin, which shows MFF is insensitive to physiologically relevant increases of mitochondrial Ca^2+^. In conclusion, the new sensor reagent MFF fluorescence is an indicator of mitochondrial chelatable Fe^2+^ in normal hepatocytes with polarized mitochondria as well as in cells undergoing loss of ΔΨ_m_.

Iron is an essential nutrient important for many biological processes. Iron also plays an important role in the generation of reactive oxygen species (ROS) by catalyzing formation of highly toxic and reactive hydroxyl radical (•OH) from hydrogen peroxide (H_2_O_2_) and superoxide (O_2_•^‒^) by the Fenton reaction (iron-catalyzed Haber–Weiss reaction) (1, 2). Iron similarly catalyzes lipid peroxidation chain reactions (3, 4). Iron mobilization into the mitochondria plays an important role in onset of the mitochondrial permeability transition and subsequent cell death in photodynamic therapy, oxidative stress, ischemia/reperfusion, and acetaminophen cytotoxicity to hepatocytes (5, 6, 7, 8, 9, 10).

Two separate pools of iron are recognized in the cytoplasm of cells: nonchelatable iron and chelatable iron (11). Nonchelatable iron includes iron tightly bound to ferritin and prosthetic groups like heme and iron-sulfur clusters, whereas chelatable iron comprises free iron and iron loosely bound to anionic metabolites and the negatively charged surfaces of membranes. Chelatable iron is redox active, cycling between the Fe^2+^ and Fe^3+^ forms, although Fe^2+^ is predominant in the cytosol and mitochondria given the reductive intracellular environment. Most chelatable iron in hepatocytes and many other cell types resides in the lysosomal/endosomal compartment, since iron uptake occurs principally by receptor-mediated endocytosis of Fe^3+^-containing transferrin (12, 13). During ischemia, the absence of an adequate ATP supply shuts down the vacuolar proton-pumping ATPase (V-ATPase) in lysosomal/endosomal membranes, which leads to lysosomal alkalinization and consequent Fe^2+^ release (7, 8). Fe^2+^ released by lysosomes enters the cytosol and is then taken up by the mitochondria (5, 6, 7, 8, 9, 10). Ferrous chelatable iron promotes mitochondrial ROS formation leading to the mitochondrial permeability transition and cell death.

Rhodamine B-[(1,10-phenanthrolin-5-yl) aminocarbonyl] benzylester (RPA) is a fluorescent indicator whose red fluorescence is quenched by Fe^2+^ (14). The delocalized positive charge of cationic RPA leads to its electrophoretic accumulation into the negatively polarized mitochondria of living cells. However, after depolarization, mitochondria release RPA. Thus, loss of mitochondrial RPA fluorescence can be due to either an increase of Fe^2+^ or, alternatively, a decrease of mitochondrial membrane potential (ΔΨ_m_). Likewise, newer iron indicators using triphenylphosphonium to target mitochondria will be sensitive to changes of ΔΨ_m_ (15, 16). Accordingly, we describe here a novel fluorescent probe to detect mitochondrial Fe^2+^, which we name mitoferrofluor (MFF). Like RPA, MFF accumulates electrophoretically into polarized mitochondria. Inside the mitochondria, MFF binds covalently to protein sulfhydryls, which prevents release of MFF after depolarization. This important feature uniquely allows monitoring of Fe^2+^ fluxes even as mitochondria depolarize.

## Results

### Spectral characteristics of MFF

**Figure 1:**
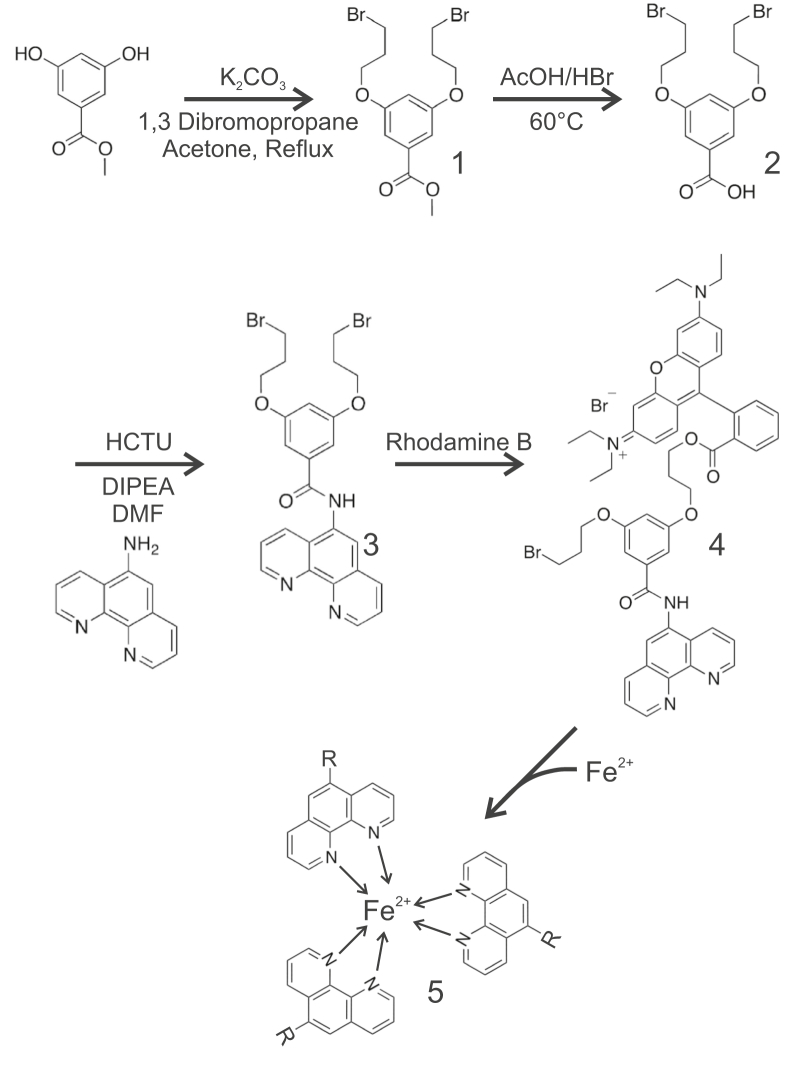
**Scheme of synthesis of mitoferrofluor.** See text for details.

**Figure 2:**
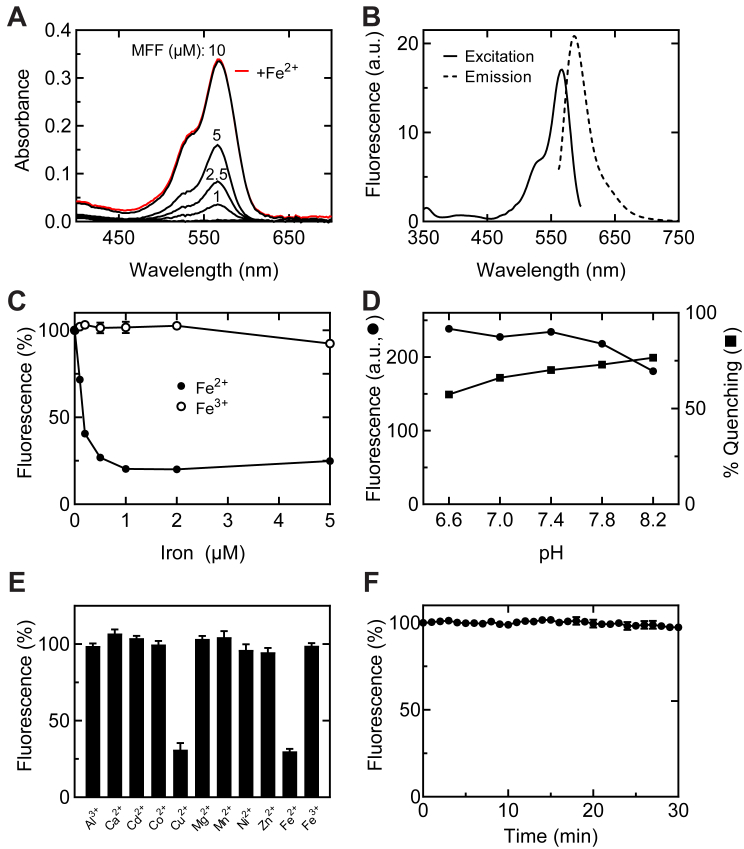
**Spectral properties and cation sensitivity of mitoferrofluor (MFF).***A*, absorbance spectra for MFF (1–10 μM) at 23 °C in 10 mM Tris–HCl containing 0.1% SDS, pH 8. *Red line* is absorbance in presence of 5 μM Fe^2+^ (FAS). *B*, excitation and emission spectra of MFF (0.5 μM) in the same medium with 600 nm and 560 nm emission and excitation, respectively, using a dual monochromator spectrofluorometer. *C*, quenching of MFF (4 μM) fluorescence by Fe^2+^ (FAS) but not Fe^3+^ (FeCl_3_). *D*, effect of pH on MFF (4 μM) fluorescence and its quenching by 1.5 μM Fe^2+^. *E*, sensitivity of MFF (4 μM) to various metal ions (1.5 μM). In panels (*C*–*E*), fluorescence was measured with a fluorescence plate reader using a 544 nm excitation filter and a 600 nm long-pass emission filter. Data points are means ± SEM from triplicate measurements. In panel (*F*), cultured mouse hepatocytes were loaded with 1 μM MFF for 30 min in complete culture medium, washed with fresh medium three times, and incubated in fresh medium for 60 min followed by 100 μM PIH. After ∼30 min, cells were placed on the stage of a Zeiss LSM880 confocal microscope, and images of MFF fluorescence were collected every 1 min for 30 min at 0.3% laser power (4 μs/pixel). Plotted is total integrated MFF fluorescence minus background as a function of time. Data points are mean ± SEM from triplicate measurements. FAS, ferrous ammonium sulfate; PIH, pyridoxal isonicotinoyl hydrazone.

MFF was synthesized as described in Experimental procedures (Fig. 1). Spectral characteristics were determined in 10 mM Tris–HCl containing 0.1% SDS at pH 8.0, which approximates the pH of mitochondria inside living cells (17, 18). The absorbance maximum of MFF was 566 nm with an extinction coefficient of 33,200 cm^−1^ M^−1^ (Fig. 2*A*). By comparison, the extinction coefficient of rhodamine B is 106,000 cm^−1^ M^−1^ at an absorbance maximum of 545 nm (19). Absorbance was linear with MFF concentration from 1 to 10 μM (Fig. 2*A*). The fluorescence excitation maximum of MFF (0.5 μM) was 567 nm, and the emission maximum was 586 nm (Fig. 2*B*).

Fluorescence of MFF (4 μM) at pH 8.0 was measured as a function of increasing Fe^2+^ (ferrous ammonium sulfate, FAS) and Fe^3+^ (ferric chloride, FeCl_3_) from freshly prepared stock solutions (1 mM). Equimolar ascorbic acid was used to maintain Fe^2+^ in the reduced state. Fe^2+^ strongly decreased the fluorescence intensity of MFF, whereas Fe^3+^ had no effect (Fig. 2*C*). Half maximal quenching occurred at 0.1 to 0.2 μM Fe^2+^. Fe^2+^ did not cause a shift of the excitation and emission spectra of MFF. Likewise, Fe^2+^ did not decrease MFF absorbance or alter the absorbance spectrum (Fig. 2*A*).

Fluorescence of MFF in the presence and absence of 1.5 μM Fe^2+^ was also measured as pH was varied between 6.6 and 8.2. MFF fluorescence in the absence of Fe^2+^ decreased slightly as pH increased, especially at pH 8.2 (Fig. 2*D*). By contrast, the percent quenching of MFF fluorescence by Fe^2+^ increased by small progressive increments as pH increased from 6.6 to 8.2. A variety of other biologically relevant multivalent cations (Al^3+^, Ca^2+^, Cd^2+^, Co^2+^, Cu^2+^, Mg^2+^, Mn^2+^, Ni^2+^, Zn^2+^) was also assessed for the ability to quench MFF fluorescence. Of these, only Cu^2+^ quenched MFF fluorescence (Fig. 2*E*). Quenching by Cu^2+^ was equal in magnitude to quenching by Fe^2+^, similar to other iron reporters like calcein and RPA (20, 21).

### MFF localizes to mitochondria

**Figure 3:**
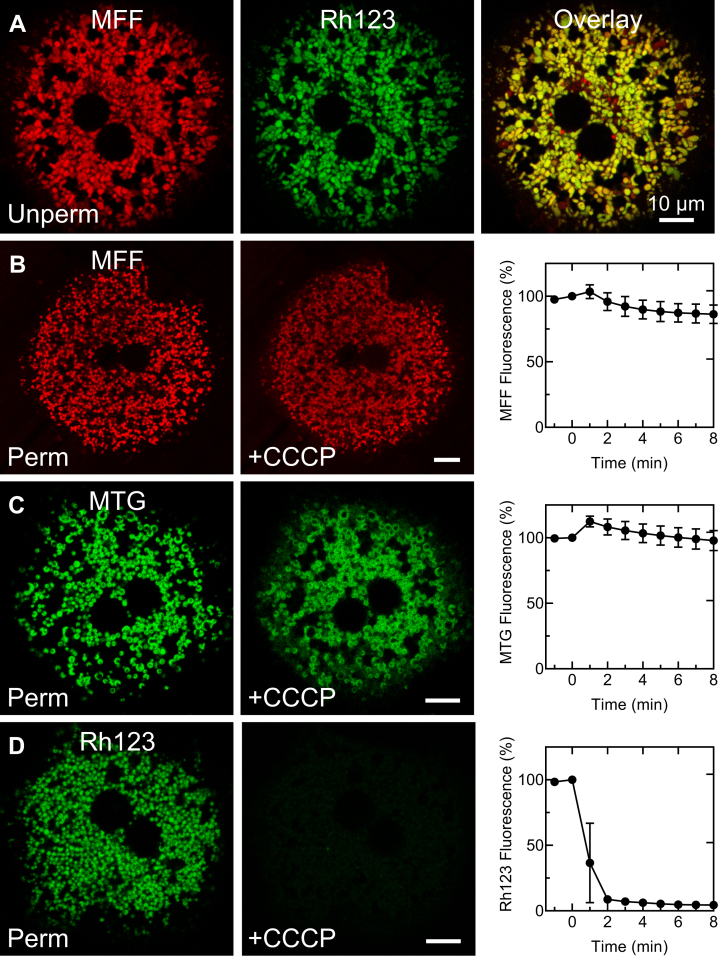
**Covalent binding of mitoferrofluor (MFF) to mitochondria.***A*, an overnight-cultured mouse hepatocyte was loaded with MFF (1 μM for 30 min), washed, and incubated in culture medium for 30 min. The hepatocyte was then loaded with Rh123 (0.5 μM) for 30 min, washed, and incubated in Krebs-Ringer-Hepes buffer (in mM: 115 NaCl, 5 KCl, 1 KH_2_PO_4_, 2 CaC1_2_, 1.2 MgSO_4_, and 25 Hepes, pH 7.4) with 150 nM Rh123 to maintain equilibrium distribution prior to confocal imaging. *B*, mouse hepatocytes were loaded with MFF (1 μM for 30 min), washed, and incubated in culture medium for 30 min. The cells were washed and incubated with supplemented ICB and 5 nM PMP to permeabilize the plasma membrane. A time series of confocal images was collected at 1 min intervals as CCCP (10 μM) was added after the second frame (0 min). Images shown are from before (*left panel*) and 2 min after (*middle panel*) addition of CCCP. The *right panel* plots *red* MFF fluorescence after background subtraction as a function of time (n = 14 cells). *C*, mouse hepatocytes were loaded with MTG (200 nM for 30 min), permeabilized, and exposed to CCCP as described in (*B*). The *right panel* plots *green* MTG fluorescence after background subtraction as a function of time (n = 12 cells). *D*, mouse hepatocytes were loaded with Rh123, as described in (*A*), followed by permeabilization and exposure to CCCP, as described in (*B*). The *right panel* plots *green* Rh123 fluorescence after background subtraction as a function of time (n = 21 cells). In (*A*), note colocalization of *red* MFF fluorescence with *green* Rh123 fluorescence, indicating mitochondrial loading of MFF. In (*B*) and (*C*), note mild mitochondrial swelling after CCCP addition, but retention of nearly all MFF and MTG fluorescence, consistent with covalent labeling of mitochondrial proteins by the fluorophores. By contrast in (*D*), Rh123 was rapidly and virtually completely released after CCCP. CCCP, carbonyl cyanide m-chlorophenyl hydrazone; ICB, intracellular buffer; MTG, MitoTracker green.

Cultured mouse hepatocytes were loaded with MFF (1 μM) for 20 min, washed three times with growth medium, and incubated for 60 min to allow MFF to form covalent adducts with mitochondrial proteins. Subsequently, the potential- and mitochondrion-indicating fluorophore, rhodamine 123 (Rh123, 0.5 μM), was added followed by imaging. As shown in Figure 3*A*, red MFF fluorescence colocalized almost perfectly with green Rh123 fluorescence. The Manders’ coefficient for colocalization of red pixels to green pixels was 0.929, whereas the Manders’ coefficient of green to red pixels was 0.996. Rarely, MFF-labeled structures did not take up Rh123 and appeared red in the overlay (Fig. 3*A*, right). These structures likely represented autophagic sequestration of mitochondria that had depolarized following MFF labeling, as previously shown after MitoTracker Green (MTG) labeling (22, 23). Thus, MFF selectively and specifically labeled mitochondria of intact cells.

### Depolarized mitochondria do not release MFF

In another experiment, mouse hepatocytes were again labeled with MFF. Cells were subsequently incubated in a high sucrose intracellular buffer containing succinate plus rotenone to support mitochondrial respiration and polarization. Agilent Seahorse XF Plasma Membrane Permeabilizer (PMP, 5 nM) was then added. Sucrose was present to minimize mitochondrial swelling that might occur after uncoupler was added. After permeabilization of the plasma membrane with PMP, mitochondria retained MFF labeling (Fig. 3*B*, left panel). After subsequent addition of carbonyl cyanide m-chlorophenyl hydrazone (CCCP, 10 μM), a protonophoric uncoupler, to depolarize mitochondria, MFF remained localized to the mitochondria (Fig. 3*B*, middle panel). A small amount of mitochondrial swelling also occurred, which spread out and diluted slightly the fluorescence of individual mitochondria. Nonetheless, total MFF fluorescence integrated within individual permeabilized hepatocytes remained nearly constant for several minutes (Fig. 3*B*, right panel). Similarly, after labeling with MTG, mitochondria retained virtually all MTG fluorescence after CCCP, although some mitochondrial swelling was again evident (Fig. 3*C*, middle panel). By sharp contrast after loading with Rh123, mitochondria of permeabilized hepatocytes released Rh123 fluorescence rapidly and virtually completely within 2 min after CCCP addition (Fig. 3*D*). Thus, MFF was well retained after mitochondrial de-energization and depolarization and to the same extent as MTG.

### MFF quenching after mitochondrial Fe^2+^ uptake

**Figure 4:**
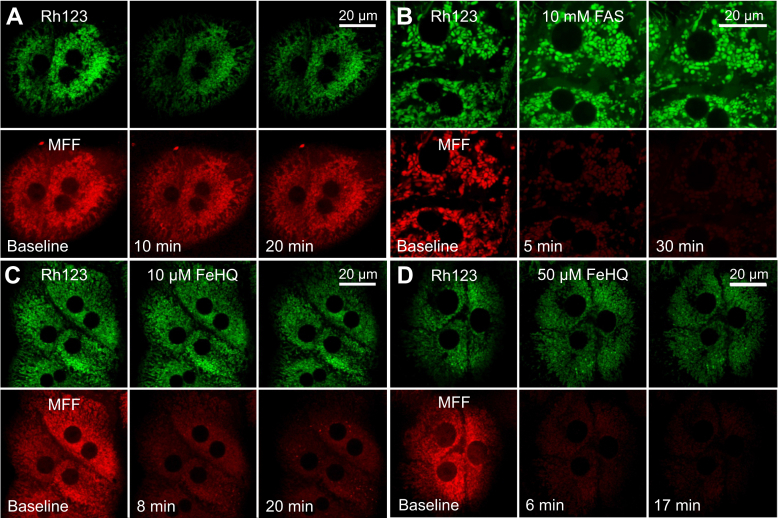
**Fluorescence quenching after iron in mitoferrofluor (MFF)-loaded hepatocytes.** Overnight-cultured rat hepatocytes were loaded with 1 μM MFF and 500 nM rhodamine 123, as described in Figure 3*A*, and then treated with vehicle (*A*), 10 mM FAS (*B*), and 10 and 50 μM FeHQ (*C* and *D*). Note marked quenching of mitochondrial MFF fluorescence after FAS and FeHQ but not after vehicle. Rh123 fluorescence was not lost. Images are representative of three or more experiments. FAS, ferrous ammonium sulfate; FeHQ, Fe3+/8-hydroxyquinoline.

Next, we determined if the fluorescence of MFF loaded into the mitochondria of living cells becomes quenched after exposure to Fe^2+^. Overnight-cultured rat hepatocytes were coloaded with MFF and Rh123, as described previously. Without further addition, Rh123 and MFF fluorescence fluctuated in parallel due to subsequently corrected microscope drift, but the ratio of MFF to Rh123 fluorescence (after background subtraction) remained constant within ±8% over 20 min of incubation (Fig. 4*A*). When Fe^2+^ was added as FAS (10 mM), MFF fluorescence decreased by 70% and 66%, respectively, after 5 and 30 min (Fig. 4*B*). In this experiment, Rh123 fluorescence imaged simultaneously increased by ∼50% after 5 and 30 min due to focus drift. Therefore, MFF/Rh123 ratios decreased by 80% and 78%.

With maintenance to the microscope and improved technique, fluctuations of MFF fluorescence over time were minimized. In the presence of the membrane-permeant transition metal iron chelator, pyridoxal isonicotinoyl hydrazone (PIH, 100 μM) (24) to chelate any endogenous iron release by proteolysis or other process, fluorescence of MFF loaded into hepatocytes was essentially constant during imaging at 1 min intervals for 30 min (Fig. 2*F*). Notably, photobleaching was absent even after prolonged imaging.

Fe^2+^ is poorly permeant through plasma membranes, and hence, high concentrations of FAS were required to quench MFF strongly. FAS at 1 mM only produced partial quenching (∼30%). Accordingly, we also exposed MFF- and Rh123-loaded hepatocytes to the membrane-permeant iron complex, Fe^3+^/8-hydroxyquinoline (FeHQ) (25). FeHQ (10 and 50 μM) also quenched mitochondrial MFF fluorescence (Fig. 4, *C* and *D*). MFF fluorescence after 10 μM FeHQ decreased by 56.4 ± 2.3% and 65.5 ± 2.4%, respectively, at 8 and 20 min (n = 3). After 50 μM FeHQ, MFF fluorescence decreased by 69.6 ± 3.1% and 81.2 ± 1.9% at 6 and 17 min, respectively (n = 3). Overall, in MFF-loaded hepatocytes, Fe^2+^ produced maximum quenching of about 80%, very similar to the quenching produced in cell-free solution (see Fig. 2*C*).

**Figure 5:**
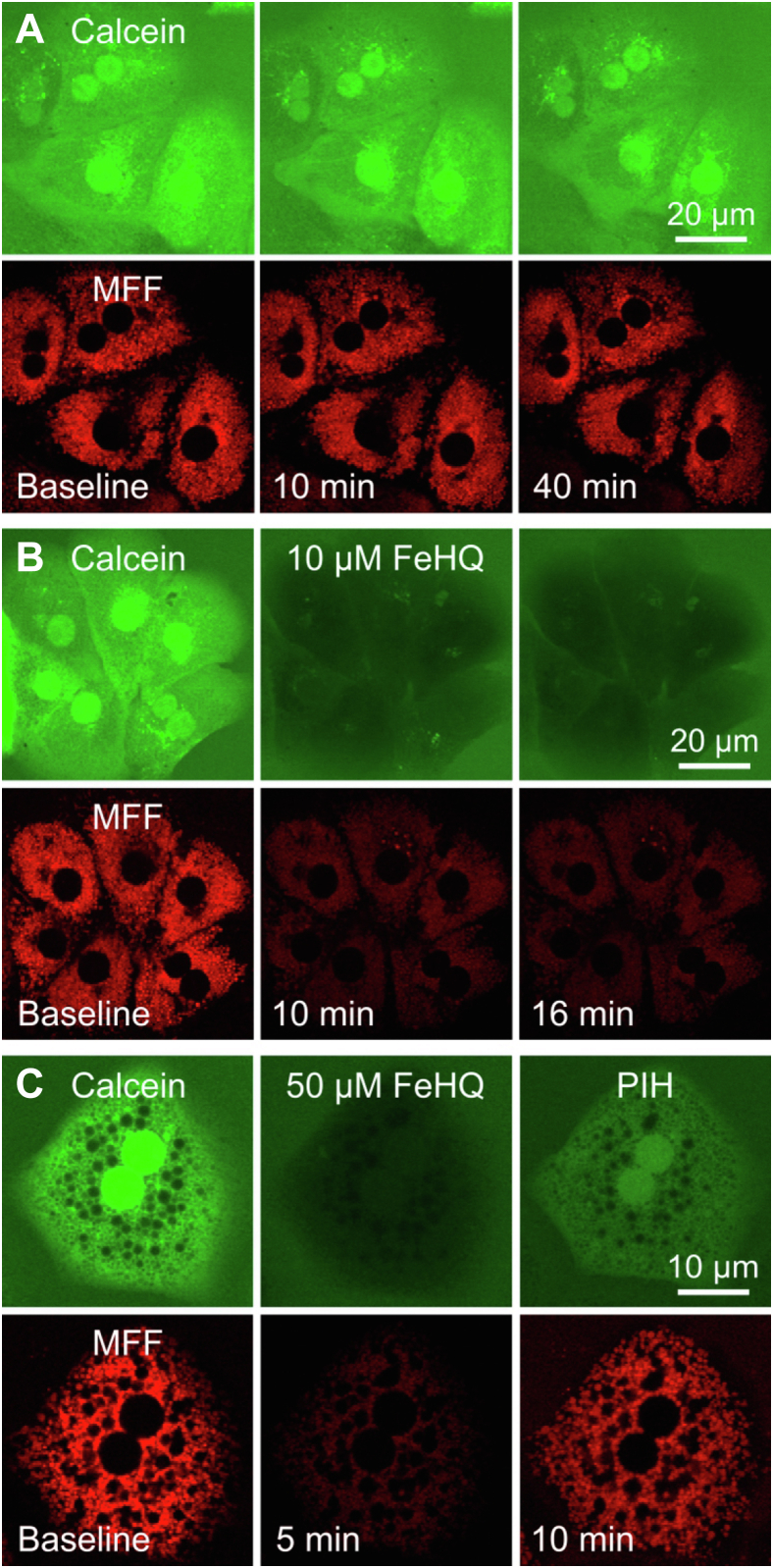
**Quenching of cytosolic calcein and mitochondrial mitoferrofluor (MFF) fluorescence by ferric 8-hydroxyquinoline: Reversal by pyridoxal isonicotinoyl hydrazone.** Overnight-cultured rat hepatocytes were loaded with 1 μM MFF and 1 μM calcein-acetoxymethylester, and then incubated with 100 μM calcein-free acid in the extracellular medium, as described in Experimental procedures. *A*, no other additions were made. *B*, 10 μM FeHQ was added as indicated. *C*, FeHQ and 1 mM PIH were sequentially added. Note that fluorescence of MFF and calcein was stable for at least 40 min (*A*). After addition of 10 μM FeHQ, both MFF and intracellular calcein became strongly quenched (*B* and *C*). Addition of PIH substantially reverted FeHQ-dependent quenching of both MFF and calcein (*C*). Images are representative of three or more experiments. FeHQ, Fe3+/8-hydroxyquinoline; PIH, pyridoxal isonicotinoyl hydrazone.

To show that Fe^3+^ is reduced to Fe^2+^ intracellularly after permeance of FeHQ, rat hepatocytes were loaded with both MFF and calcein-acetoxymethylester. Calcein-free acid was also placed in the extracellular medium as an internal reference. Over 40 min of incubation, extracellular calcein fluorescence remained virtually unchanged, whereas intracellular MFF and calcein fluorescence declined slightly by 20% and 16%, respectively, perhaps due to spontaneous intracellular iron release (Fig. 5*A*). After FeHQ addition, both MFF and intracellular calcein fluorescence became strongly quenched and decreased by 70.9 ± 1.8% and 71.6 ± 2.7%, respectively, at 16 min after 10 μM FeHQ, and by 77.3 ± 2.6% and 81.1 ± 1.9%, respectively, at 5 min after 50 μM FeHQ (n = 3 for each concentration) (Fig. 5, *B* and *C*). Since Fe^2+^ but not Fe^3+^ quenches green calcein fluorescence (9, 11, 20), these findings directly documented intracellular reduction of Fe^3+^ contained in FeHQ to Fe^2+^. By contrast, fluorescence of calcein-free acid placed in the extracellular medium persisted in all experiments within a range of ±12%. Subsequent addition of the membrane-permeant transition metal iron chelator, PIH, substantially reverted FeHQ-dependent quenching of both MFF and calcein (Fig. 5*C*, see also Fig. 7). Reversal was incomplete because of competition between PIH and MFF or calcein for binding of Fe^2+^.

### Ca^2+^ does not quench MFF fluorescence

**Figure 6:**
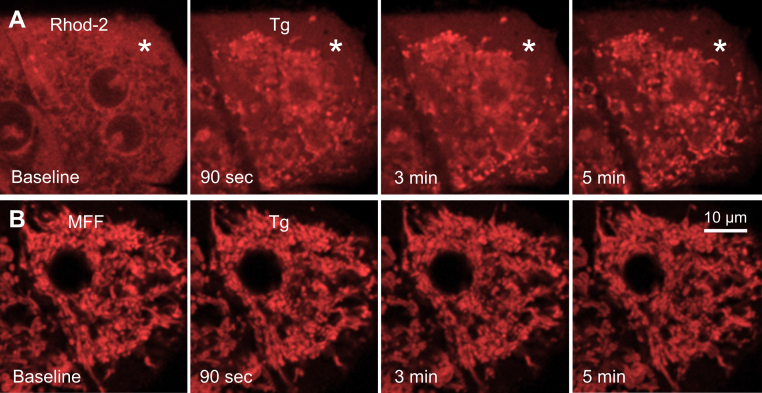
**Lack of response of mitoferrofluor (MFF) to increased mitochondrial Ca**^**2+**^**.** In separate experiments, overnight-cultured rat hepatocytes were loaded with 10 μM Rhod-2 AM (*A*), a red-fluorescing Ca^2+^ indicator, and 1 μM MFF (*B*). Thapsigargin (Tg, 2 μM) was then added. In (*A*), note a time-dependent increase in mitochondrial Rhod-2 fluorescence in a heterogeneous fashion. The *asterisk* identifies an area of diffuse Rhod-2 fluorescence that decreased after thapsigargin. In (*B*), MFF fluorescence remained virtually unchanged after thapsigargin, and heterogeneous changes of MFF fluorescence were completely absent. Images are representative of three or more experiments.

To exclude the possibility that MFF responds to physiologically relevant increases of Ca^2+^, we treated rat hepatocytes with thapsigargin (2 μM), an inhibitor of the smooth endoplasmic reticulum (ER) calcium-pumping ATPase (SERCA) (26). Thapsigargin leads to release of Ca^2+^ from the ER, some of which moves into the mitochondria (27). In a first set of experiments, we loaded hepatocytes with Rhod-2, a Ca^2+^ indicator. Before thapsigargin addition, mitochondria were dark voids surrounded by diffuse patches of red fluorescence (Fig. 6*A*, baseline). After thapsigargin, most mitochondria showed a marked increase in Rhod-2 fluorescence beginning within 90 s, signifying mitochondrial Ca^2+^ uptake. Notably, mitochondrial Ca^2+^ uptake was heterogeneous with some mitochondria taking up Ca^2+^ at earlier times than others. At the same time, diffuse cytoplasmic patches of Rhod-2 fluorescence declined considerably, suggesting that the diffuse patches were ER that released Ca^2+^ after thapsigargin treatment, which then entered mitochondria, at least in part (Fig. 6*A*, asterisks). Also, before thapsigargin, rings of Rhod-2 fluorescence surrounding nuclei were present that disappeared after thapsigargin. These rings are nuclear membranes, which are extensions of the ER that likewise released their calcium content after thapsigargin.

In parallel experiments, we loaded hepatocytes with MFF. After thapsigargin addition, MFF fluorescence as a percentage of baseline fluorescence was 98%, 93%, and 92%, respectively, after 90 s, 3 min and 5 min, which was comparable to decreases occurring in the absence of added thapsigargin (Fig. 6*B*, compare to Fig. 5*A*). During this time, mitochondria became well loaded with Ca^2+^ (see Fig. 6*A*). Slight changes in overall MFF brightness occurring at different time points after thapsigargin were likely attributable to small changes in the focal plane or spontaneous Fe^2+^ release rather than to an effect of Ca^2+^, since heterogeneous changes of mitochondrial MFF fluorescence over time were not evident. Overall, the results show that intracellular MFF fluorescence is specific for Fe^2+^ and does not respond to increases of mitochondrial Ca^2+^, consistent with Figure 2*E*.

### MFF responds to mitochondrial iron in cell lines

**Figure 7:**
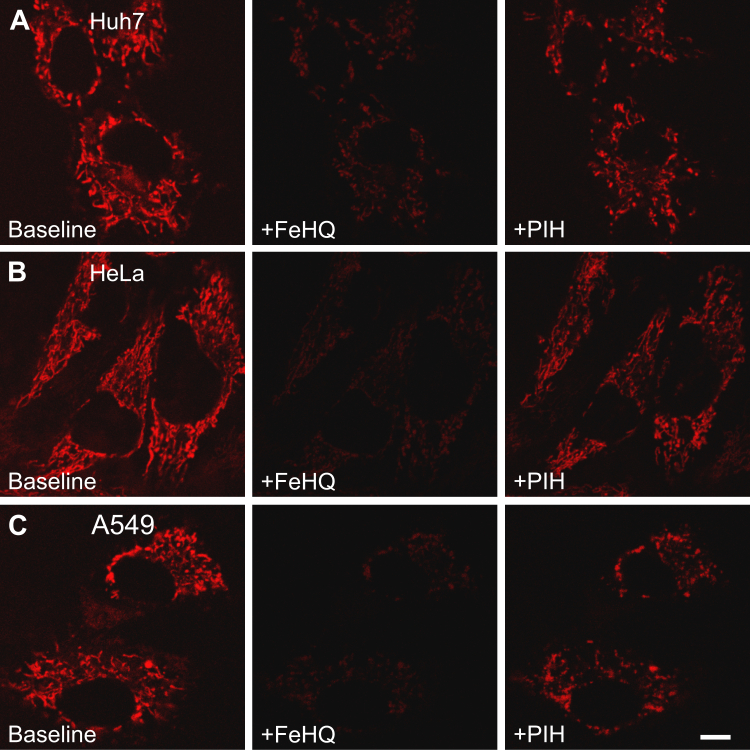
**Iron quenches mitoferrofluor (MFF) fluorescence in mitochondria of cultured cell lines.** Huh7, HeLa, and A549 cells (*A–C,* respectively) were loaded with 1 μM MFF for 30 min in culture medium and then thrice washed with medium. After ∼60 min more of incubation, the cells were placed on the microscope, and the red fluorescence of MFF was imaged before (Baseline) and after sequential addition of 50 μM FeHQ and 100 μM PIH at 5 min intervals. Note, quenching of MFF fluorescence after FeHQ and partial recovery of fluorescence after PIH in each cell type. Images are representative of three or more experiments. The scale bar represents 10 μm. FeHQ, Fe3+/8-hydroxyquinoline; PIH, pyridoxal isonicotinoyl hydrazone.

MFF was also loaded into Huh7 human hepatocarcinoma cells, HeLa human cervical cancer cells, and A549 human pulmonary adenocarcinoma cells (Fig. 7). In each cell line, red MFF fluorescence showed a characteristic mitochondrial distribution, which became quenched after 50 μM FeHQ. Quenching at 5 min after FeHQ was 78.9 ± 2.8%, 83.4 ± 2.4%, and 84.8 ± 2.6%, respectively, for Huh7, HeLa, and A549 (n = 6 cells per line). Subsequent iron chelation with PIH partially reversed MFF quenching to 57.6 ± 2.5%, 53.6 ± 6%, and 46.4 ± 2.9%, respectively, showing that the fluorescence signal of MFF was iron dependent (Fig. 7).

### MFF does not alter hepatocyte respiration

**Figure 8:**
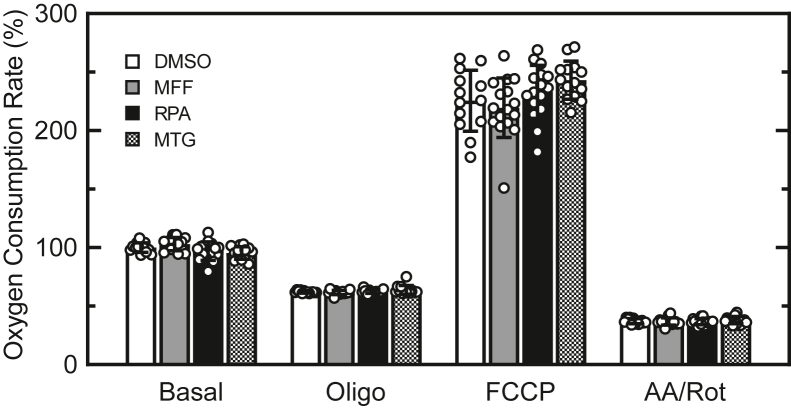
**Mitoferrofluor (MFF), RPA, and MitoTracker green do not change mitochondrial respiration.** Overnight-cultured rat hepatocytes were loaded with dimethyl sulfoxide (DMSO, vehicle), 1 μM MFF, 1 μM RPA, or 1 μM MTG. OCR was measured before (Basal) and after sequential addition of oligomycin (1 μM), FCCP (1 μM), and antimycin/rotenone (1 μM each) with a Seahorse XF^e^96 extracellular flux analyzer. Error bars are SD. MTG, MitoTracker Green; OCR, oxygen consumption rate; RPA, rhodamine B-[(1,10-phenanthrolin-5-yl)aminocarbonyl]benzyl ester.

Cultured rat hepatocytes were loaded with MFF (binds covalently *via* halomethyl group), RPA (mitochondrially targeted iron indicator that does not bind covalently), or MTG (mitochondrially targeted fluorophore that binds covalently like MFF), all at a concentration of 1 μM. Subsequently, oxygen consumption rates (OCRs) were measured before and after sequential addition of oligomycin (ATP synthase inhibitor), carbonyl cyanide trifluoromethoxyphenylhydrazone (FCCP, uncoupler), and antimycin/rotenone (respiratory inhibitors) in a Seahorse XF^e^96 Extracellular Flux Analyzer (mitochondrial stress test). OCRs were then expressed as the percent of basal respiration by vehicle (dimethyl sulfoxide)-treated hepatocytes just before addition of oligomycin. Correcting for nonmitochondrial antimycin A/rotenone-insensitive OCR, oligomycin inhibited basal mitochondrial OCR by 60%, whereas as FCCP increased OCR by 200% (Fig. 8). Neither MFF, RPA nor MTG altered any of these OCRs.

## Discussion

Chelatable iron is highly important in physiological and pathophysiological processes (28, 29, 30, 31). For example, mitochondrial chelatable iron contributes to hepatocellular injury from ischemia/reperfusion, acetaminophen hepatotoxicity, and oxidative stress (5, 6, 7, 8, 9, 10, 32, 33). Compared to Ca^2+^, technologies to measure mitochondrial Fe^2+^ are less well developed. Measurements of total iron and radioactive ^56^Fe are frequently used to assess mitochondrial iron content in cells and tissues, but such approaches require subcellular fractionation and purification of mitochondria during which iron, especially chelatable iron, may be lost (34). Fluorescent iron sensors permit measurements of chelatable iron *in situ* in living cells (11, 14, 15, 16, 35). Previously, we developed a cold loading/warm incubation protocol to ester-load calcein into the mitochondria, and we showed in rat hepatocytes that green calcein fluorescence became quenched after various treatments that mobilize Fe^2+^ from lysosomes (7, 9). Although Cu^2+^ also quenches calcein fluorescence, the quenching in these examples was prevented by desferal and starch-desferal, chelators that bind iron but not copper. Nonetheless, the cold loading/warm incubation protocol has species and cell type specificities. In particular, cold ester loading/warm incubation localizes several fluorophores into mitochondria of rat hepatocytes and myocytes but fails to localize the same fluorophores into the mitochondria of mouse hepatocytes.

RPA is a red-fluorescing probe that was designed specifically to monitor mitochondrial Fe^2+^ in living cells by confocal microscopy (14). Cationic RPA accumulates electrophoretically in response to the negative ΔΨ_m_ of polarized mitochondria. Inside the mitochondria, RPA becomes quenched as Fe^2+^ is taken up. However, mitochondrial depolarization causes release of cationic RPA, which also leads to a decrease of mitochondrial RPA fluorescence. Normal ΔΨ_m_ in hepatocytes is −120 to −150 mV, and even a 20 mV decrease in the magnitude of ΔΨ_m_ will lead to a >50% loss of permeant monovalent cations like RPA. Thus, decreased mitochondrial RPA fluorescence does not distinguish increased mitochondrial Fe^2+^ from decreased ΔΨ_m_ unless ΔΨ_m_ is monitored independently. More recently, mitochondrially targeted cationic iron probes have been developed that increase fluorescence by an Fe^2+^-catalyzed oxidation (15, 16). Such probes will also be released by mitochondria after membrane depolarization.

Accordingly, we developed MFF to detect mitochondrial Fe^2+^ separately of changes of ΔΨ_m_. MFF was synthesized from three chemical components: (1) rhodamine B, which provides both red fluorescence and a delocalized positive charge; (2) aminophenanthroline, which chelates Fe^2+^ in the ratio of three aminophenanthrolines to one Fe^2+^; and (3) a linker containing a reactive bromomethyl group that forms covalent adducts with protein thiols (Fig. 1). Like RPA, MFF accumulates electrophoretically into polarized mitochondria, and its red fluorescence colocalizes with ΔΨ_m_-indicating Rh123 (Fig. 3*A*). However, unlike RPA, MFF binds covalently to mitochondrial matrix proteins, which prevents release of MFF after depolarization (36, 37). Thus, the mitochondria retained MFF after depolarization when Rh123 was completely lost (Fig. 3, *B* and *D*). This important feature uniquely allows monitoring of Fe^2+^ fluxes even as mitochondria become dysfunctional (6). Based on our experience with MitoTracker dyes, covalent adduction of halomethyl moieties to mitochondrial proteins is not particularly rapid (37, 38). Thus, after the initial electrophoretic mitochondrial uptake of MFF, we further incubated cells in the absence of exogenous MFF for 30 to 60 min to allow adduct formation to go to completion.

Similar to RPA, MFF fluorescence has excitation and emission maxima at 567 and 586 nm, respectively (Fig. 2*B*). In cell-free solution, this fluorescence was quenched by Fe^2+^ but not by Fe^3+^ to a maximal extent of 75% to 80% at pH 7.8 to 8.2, the pH of the mitochondrial matrix (Fig. 2, *C* and *D*). After loading into mitochondria of hepatocytes, MFF fluorescence was also strongly quenched by Fe^2+^ to a maximal extent of ∼80%, as shown by decreased red MFF fluorescence after addition of FAS and membrane-permeant FeHQ (Figs. 4 and 5). Similar iron-induced quenching also occurred in three different cancer cell lines (Fig. 7). Importantly, MFF was photostable under the imaging conditions used (Fig. 2*F*).

Because of the poor permeance of Fe^2+^, a high extracellular concentration of FAS, 10 mM, was required to achieve maximal MFF quenching. By contrast, a much lower concentration of FeHQ (10–50 μM) caused comparable MFF quenching (Figs. 4 and 5). FeHQ is a membrane-permeable chelate of Fe^3+^ (25), but once inside hepatocytes, Fe^3+^ became reduced to Fe^2+^, at least in part, as shown by FeHQ-dependent quenching of intracellular but not extracellular calcein (Fig. 5). Subsequent addition of the iron chelator PIH caused recovery of fluorescence, which showed the reversibility of Fe^2+^-dependent quenching of MFF.

Ca^2+^ is an important modulator of mitochondrial and cellular function and is also a regulator of cell death (39, 40, 41). To determine whether Ca^2+^ altered mitochondrial MFF fluorescence *in situ*, we exposed hepatocytes to thapsigargin, a SERCA inhibitor (26). As expected, thapsigargin led to Ca^2+^ release from the ER and Ca^2+^ uptake into the mitochondria, as revealed by Rhod-2 fluorescence, which decreased in patchy putative areas of ER and increased sharply and heterogeneously in punctate mitochondria (Fig. 6). By contrast, MFF fluorescence remained unchanged after thapsigargin. Thus, MFF was insensitive to physiologically relevant changes of mitochondrial Ca^2+^. Likewise, MFF was insensitive to other biologically relevant divalent cations, except for Cu^2+^, which also quenched MFF fluorescence (Fig. 2*E*).

Like RPA and other iron indicators, MFF measures relative changes in Fe^2+^ (20, 42, 43). Assuming near equilibrium uptake of MFF into polarized mitochondria, MFF likely accumulates to concentrations of 100 μM or more inside mitochondria of living cells under our loading conditions. For this reason, MFF fluorescence is likely less sensitive to changes of physiological chelatable iron estimated to be 15 to 20 μM in mitochondria (42) than to pathophysiologic iron overload. Accordingly, MFF may be more useful to monitor iron loading under pathophysiological conditions. Recently, while the present paper was in revision, our group used MFF to show that mitochondrial iron uptake and overload leads to ROS formation during acetaminophen toxicity in cultured hepatocytes with consequent onset of the mitochondrial permeability transition and cell death, which illustrates the utility of MFF as a mitochondrial Fe^2+^ probe (6). Additional studies will be needed to characterize the advantages and limitations of using of MFF to characterize changes of mitochondrial chelatable iron in different physiological and pathophysiological contexts.

A concern was whether MFF at the concentrations used would alter mitochondrial function because of its covalent binding to mitochondrial proteins. However, measurements of basal OCR and OCR after sequential addition of oligomycin, FCCP and antimycin/rotenone showed no differences compared to vehicle-treated hepatocytes (Fig. 8). Likewise, MTG and RPA did not alter coupled respiration and maximal respiratory capacity.

In conclusion, MFF is a new red-fluorescing indicator of mitochondrial chelatable Fe^2+^ in living cells. Importantly, MFF forms protein adducts inside mitochondria such that MFF is retained in a ΔΨ_m_-independent fashion. This characteristic makes MFF useful to monitor mitochondrial iron under pathological conditions when mitochondrial depolarization may occur.

## Experimental procedures

### Synthesis of MFF

#### General

Reactions were monitored by analytical TLC or using an Agilent 6120 quadrapole LC/MS system. Flash chromatography purification was done with a Teledyne ISCO system on either silica or C-18 support. All reactions were carried out under argon at a slight positive pressure except where noted. ^1^H-NMR spectra were recorded at 400 MHz on a Bruker spectrometer. ^13^C-NMR spectra were recorded at 100 MHz, and all chemical shifts were reported from the solvent resonance of CDCl_3_ (7.27 ppm ^1^H-NMR, 77.23 ^13^C-NMR) or D6-dimethyl sulfoxide (2.5 ppm ^1^H-NMR, 39.5 ^13^C-NMR). Reverse phase analytical runs used a 95:5 water (0.1% formic acid)/acetonitrile–100% acetonitrile gradient over 10 min with a 3 min 100% acetonitrile wash on a 20 × 4.6 mm Phenomenex Luna 3-μm C18 100 Å pore column at a flow rate of 1.0 ml/min.

#### Methyl 3,5-bis(3-bromopropoxy)benzoate

To an oven-dried flask equipped with a reflux condenser that was cooled under an argon atmosphere was added methyl 3,5-dihydroxybenzoate (2.0 g, 11.9 mmol), potassium carbonate (4.11 g, 29.8 mmol), and 0.2 l of anhydrous acetone. After stirring at room temperature (RT) for 10 min, 1,3 dibromopropane (12.0 ml, 119 mmol) was added, and the solution was heated to 70 °C. The reaction continued to stir at this temperature overnight. The next day, the reaction was cooled to RT, concentrated in a rotary evaporator, taken up in approximately 100 ml of dichloromethane, filtered, and concentrated. Purification was done using flash chromatography on silica with hexanes/ethyl acetate (9:1) to afford the product methyl 3,5-bis(3-bromopropoxy)benzoate (Fig. 1, Structure 1) (2.47 g, 51% yield).

#### 3,5-Bis(3-bromopropoxy)benzoic acid

To a clean dry flask equipped with an air-to-air condenser was added methyl 3,5-bis(3-bromopropoxy) benzoate (2.47 g, 6.01 mmol), as synthesized previously, followed by acetic acid/HBr (2:1 by weight, 12 ml) with constant stirring. The solution was heated to 60 °C and stirred overnight. The next day, the solution was cooled to 0 °C to form a precipitate, 3,5-bis(3-bromopropoxy)benzoic acid (Fig. 1, Structure 2). The precipitate was collected by filtration, rinsed two times with hexanes, dried under vacuum, and used without any further purification. The identity of the product was confirmed by mass spectrometry giving an observed mass of 392 amu for the M-1 ion.

#### 3,5-Bis(3-bromopropoxy)-N-(1,10-phenanthrolin-5-yl)benzamide

To a flame-dried flask cooled under argon was added 3,5-bis(3-bromopropoxy)benzoic acid (0.79 g, 2.0 mmol), as synthesized previously, and 5-aminophenanthroline (0.39 g, 2.0 mmol). The two compounds were then taken up in 20 ml of anhydrous dimethyl formamide and O-(6-chlorobenzotriazol-1-yl)-N,N,N′,N′-tetramethyluronium hexafluorophosphate (0.91 g, 2.2 mmol) was added. After 5 min, diisopropylethylamine (1.1 ml, 6.0 mmol) was added. After 5 days of stirring at RT, the reaction was concentrated in a rotary evaporator, taken up in ethyl acetate, and washed with saturated sodium bicarbonate. Upon removal of the organic layer, the aqueous layer was saturated with sodium chloride and washed twice more with ethyl acetate to remove residual product trapped in the aqueous phase. Confirmation of removal of residual product in the aqueous phase was done by LC/MS of the aqueous phase. Purification was done *via* C18 reverse phase chromatography using a 95:5 water (0.1% formic acid)/acetonitrile–100% acetonitrile gradient to afford the product (Fig. 1, Structure 3). The approximate yield was 20%, and the identity was confirmed by mass spectrometry giving an observed mass of 572 amu for the M + 1 ion.

#### N-(9-(2-((3-(3-((1,10-phenanthrolin-5-yl)carbamoyl)-5-(3-bromopropoxy)phenoxy) propoxy)carbonyl)phenyl)-6-(diethylamino)-3H-xanthen-3-ylidene)-N-ethylethanaminium bromide

To a flame-dried flask was added 3,5-bis(3-bromopropoxy)-N-(1,10-phenanthrolin-5-yl)benzamide (0.022 g, 0.038 mmol), as synthesized previously, and rhodamine B base (0.0184 g, 0.038 mmol), followed by 0.4 ml anhydrous dimethyl formamide. The solution was then heated to 100 °C and stirred overnight. The next day, the solution was concentrated in a rotary evaporator, taken up in 0.8 ml of methanol, and purified *via* C18 reverse phase chromatography using a 95:5 water (0.1% formic acid)/acetonitrile–100% acetonitrile gradient to afford the final product (Fig. 1, Structure 4). The approximate yield was 10%, and the identity was confirmed by mass spectrometry giving an observed mass of 935 amu for the M + 1 ion. The final product we named as MFF. Fe^2+^ binds the phenanthroline portion of MFF to form a 1:3 metal–ligand complex (Fig. 1, Structure 5). Dissolved in dimethyl sulfoxide, MFF was stable for at least 3 months at −20 °C.

### Absorbance and fluorescence spectra measurements

Absorbance and fluorescence spectra of MFF in 10 mM Tris–HCl buffer containing 0.1% SDS, pH 8.0 were obtained at 23 °C using a Shimadzu UV-1800 UV-VIS Spectrophotometer and a Photon Technology International Quanta Master spectrofluorometer (HORIBA Scientific), respectively. Quenching of MFF (4 μM) by FAS and FeCl_3_ was measured using a NOVOstar fluorescence plate reader (BMG Labtech). Aqueous FAS was prepared with equimolar ascorbic acid to prevent oxidation. Tris buffer was treated with Chelex (1421253, BioRad) overnight to remove trace heavy metals. Fluorescence spectra were uncorrected for lamp intensity and detector sensitivity.

### Hepatocyte isolation and culture

Primary hepatocytes were isolated from overnight-fasted male Sprague–Dawley rats (200–250 g body weight) and C57BL/6 mice (20–25 g) by collagenase perfusion, as described previously (7, 9). Hepatocytes were plated on type 1 collagen–coated glass bottom MatTek 35-mm dishes at a density of 300,000 cells per dish and cultured overnight in Waymouth's MB-742/1 growth medium (11220035, Thermo Fisher Scientific) containing 27 mM NaHCO_3_, 2 mM L-glutamine, 10% fetal calf serum (FCS) (S12450, R&D Systems), 100 nM insulin (NDC 0169-1833-11, Novo Nordisk), and 10 nM dexamethasone (NDC 67457-422-54, Viatris), pH 7.4 at 37 °C in 5% CO_2_/air, unless otherwise indicated. All animals were given humane care using protocols approved by the Animal Care and Use Committee of the Medical University of South Carolina.

### Loading of fluorophores

Hepatocytes were loaded with 1 μM MFF for 20 min, washed three times with growth medium, and then incubated for 60 min before microscopy. In some experiments, cells were loaded or coloaded with 200 to 500 nM Rh123 (R302, Thermo Fisher Scientific), 200 nM MTG (M7514, Thermo Fisher Scientific), 1 μM RPA (ME043, Squarix Biotechnology), or 1 μM calcein-acetoxymethylester (C3100MP, Thermo Fisher Scientific). After Rh123 or calcein coloading, imaging was performed in the presence of ∼1/3 of the initial loading Rh123 concentration or 100 μM calcein-free acid, respectively. To monitor mitochondrial calcium uptake, hepatocytes plated for 5 to 6 h were loaded with 10 μM Rhod-2 AM (R1245MP, Thermo Fisher Scientific) for 1 h in growth medium followed by three washes with medium. All loading was carried out at 37 °C.

### Confocal microscopy and image analysis

Green and red fluorescence was imaged with an inverted Zeiss LSM510 or LSM880 laser scanning confocal microscope using a 63× NA 1.4 oil-immersion plan apochromat lens or an Olympus FV10i confocal microscope using a 60× water immersion super apochromat objective. During imaging, cells were maintained in a humidified chamber at 37 °C in 5% CO_2_/air. Images shown are representative of three or more experiments. Fluorescence was quantified using Adobe Photoshop CS4 and Zeiss Zen software. To quantify MFF fluorescence, cells were outlined, and mean fluorescence intensity was determined by histogram analysis of the red channel. Background values were obtained from images collected while focusing within the coverslip and subtracted from mean fluorescence of each field. To prepare FeHQ, 29 mg of HQ (252565, Sigma) was dissolved in 10 ml of dimethylsulfoxide to make a 20 mM 8-HQ solution. With stirring, 27 mg of FeCl_3_ hexahydrate (10 mM, 31232, Sigma) was added to make the FeHQ complex solution. For plasma membrane permeabilization, hepatocytes were washed and incubated in intracellular buffer (250 mM sucrose, 10 mM NaCl, 1 mM KH_2_PO_4_, 20 mM Tris, 5 mM MgCl_2_, 5 mM succinic acid, 0.2% bovine serum albumin, pH 7.2) supplemented with 2 μM NIM811 (Novartis), 2 μM rotenone (557368, CalBiochem), 5 μg/ml oligomycin A (75351, Sigma), 2 μM thapsigargin (T9033, Sigma), and 2 μM N,N′-diphenyl-p-phenylenediamine (292265, Sigma). PMP (102504-100, Agilent) was then added at a final concentration of 5 nM.

### Measurement of hepatocyte respiration

Isolated rat hepatocytes were plated on type I collagen–coated Seahorse XF96 microplates at a density of 10,000 cells per well and cultured in Waymouth's MB-742/1 growth medium containing 27 mM NaHCO_3_, 2 mM L-glutamine, 10% FCS, 100 nM insulin, and 10 nM dexamethasone, pH 7.4 at 37 °C in 5% CO_2_/air incubator. The following day, hepatocytes were treated with 1 μM MFF, 1 μM RPA, 1 μM MTG, or vehicle for 20 min in the culture medium. Subsequently, the culture medium was replaced with assay buffer which contained 130 mM NaCl, 5.3 mM KCl, 1.8 mM CaCl_2_, 0.6 mM MgCl_2_, 0.5 mM KH_2_PO_4_, 0.5 mM Na_2_HPO_4_, 4 mM glutamine, 5.6 mM glucose, 2 mM pyruvate, 100 nM insulin, 0.1% bovine serum albumin, 0.1% MEM nonessential amino acids (11140050, Gibco), 0.1% MEM amino acids (11130051, Gibco), 0.1% MEM vitamins, and 1% FCS. Hepatocytes were allowed to equilibrate in the assay buffer at 37 °C without CO_2_ for 1 h before OCR was measured in a Seahorse Bioscience XF^e^96 Extracellular Flux Analyzer.

### Statistical analysis

Data are presented as means ± SEM unless otherwise indicated. Statistical significance was determined by Student *t* test using *p* < 0.05 as the criterion. All statistical procedures were performed using the Sigma Plot statistical software package (Systat Software). Sample sizes were three or greater. Images shown are representative of three or more experiments.

## Data availability

All data generated and analyzed during this study are available from the corresponding author on reasonable request.

## Conflict of interest

The authors declare that they have no conflicts of interest with the contents of this article.
